# The Gut Microbiota of Peruvian Children Under the Age of Two During the Complementary Feeding Period

**DOI:** 10.3390/ijerph22091369

**Published:** 2025-08-30

**Authors:** Ayat Kutbi, Junming Gong, Douglas Dluzen, Mihai Pop, Yuejin Li

**Affiliations:** 1 Department of Biology, School of Computer, Mathematical, and Natural Sciences, Morgan State University, Baltimore, MD 21251, USA; 2 Department of Epidemiology and Environmental Health, School of Public Health and Health Professions, University at Buffalo, The State University of New York, Buffalo, NY 14214, USA; junmingg@buffalo.edu; 3 Department of Computer Science, College of Computer, Mathematical, and Natural Sciences, University of Maryland, College Park, MD 20742, USA; mpop@umd.edu

**Keywords:** microbiota, gut microbiota, breastfeeding, complementary foods, MAL-ED, Peru

## Abstract

The gut microbiota plays a crucial role in early childhood development. Dysbiosis in this community has been linked to risk of disease. The transition from an exclusive milk-based diet to complementary feeding and eventual weaning is crucial for the development and maturation of the gut microbiota. However, the impact of breastfeeding duration during the complementary feeding period (typically 6 to 24 months of age) on microbial development trajectories remains incompletely characterized. Here, we investigated whether prolonged breastfeeding during the complementary feeding window influences gut microbiota succession by comparing children who continued breastfeeding with those who were fully weaned at the same ages. We analyzed 16S rRNA gene sequencing profiles of fecal samples collected from a cohort of Peruvian children at 6, 12, 18, and 24 months of age. Samples were categorized into two groups: a complementary feeding group (BF), which received both complementary foods and breast milk, and a post-weaning group (NBF), which had stopped receiving breast milk for over 7 days. We conducted both alpha and beta diversity to assess within- and between-sample microbial variation. Relative abundances of microbial taxa at the phylum and genus levels were also quantified. The results showed a clear age-related increase in both species richness and species evenness across early childhood, while BF had more gradual changes relative to NBF. Marked differences in community composition were observed between BF and NBF children at 12, 18, and 24 months, respectively. Children who continued breastfeeding maintained a *Bifidobacterium*-rich, lower-diversity microbiota for a longer period, whereas weaned children at the same age exhibited accelerated microbiota development toward an adult-like profile enriched in Bacteroidota and Bacillota (formerly Firmicutes). These findings suggest that breastfeeding during the complementary feeding period exerts a significant influence on gut microbiota development through the first two years of life, which is most likely independent of complementary food. The study provides potential implications for children’s health and nutrition guidelines from a perspective of gut microbiota succession.

## 1. Introduction

The gut microbiota plays a fundamental role in human health, beginning early in life when it influences immune system maturation, nutrient absorption, and protection against pathogens [1,2]. During this critical developmental window, disruptions in microbial colonization can alter growth trajectories and immune tolerance development [3,4]. Emerging evidence indicates that these early-life microbial patterns not only impact immediate health outcomes but may also have lasting effects on long-term metabolic, immunological, cardiovascular, and neurological function [5,6,7,8,9]. Understanding how gut microbiota develops and interacts with host physiology in early life is therefore essential for informing early interventions to promote lifelong health.

At birth, the neonatal gut is nearly sterile and quickly acquires microbes from the maternal vagina, skin, gut, breast milk, and environment; the composition and abundance of these microbes are primarily influenced by maternal, dietary, clinical and pharmacological factors, for example, mode of delivery and early feeding practices [10,11,12]. In these very early days, the neonatal gut microbiota is colonized by facultative anaerobes, such as *Escherichia*. By the end of the first week, obligate anaerobic genera, including *Bifidobacterium*, *Bacteroides*, and *Clostridium*, begin to expand, coinciding with the establishment of an anaerobic gut environment and early feeding [13,14]. Among them, *Bifidobacterium* become the most abundant, particularly in breastfed infants due to their unique capacity to metabolize human milk oligosaccharides (HMOs). The initial microbial seeding establishes a dynamic ecosystem within the infant gut, promoting further gut microbiota succession.

It is widely accepted that human breast milk provides a complex combination of nutrients and bioactive components to support growth, immune protection, neurological development, and the establishment of a healthy gut microbiota [15,16,17,18]. Infants receiving human breast milk foster a distinct a distinct and beneficial gut microbial profile, characterized by lower diversity but a higher prevalence of beneficial bacteria, such as *Bifidobacterium* and *Lactobacillus*, which are associated with immune system development, healthier metabolic pathways, and better health outcomes compared to those introduced to other foods or liquids earlier [17,18,19].

As children transition from exclusive breastfeeding to complementary feeding with liquid and solid foods during the weaning period, and eventually to the cessation of breastfeeding upon completion of weaning, the gut microbiota undergoes a marked increase in both diversity and complexity [20]. Food introduction is a primary contributor to the characterized increase in alpha diversity during complementary feeding, because it introduces new dietary substrates that favor the expansion of food-associated taxa, such as *Bacteroides* and fiber-degrading obligate anaerobes, including *Faecalibacterium*, *Ruminococcus*, *Blautia*, and *Roseburia* [21,22]. In parallel, the cessation of breastfeeding contributes independently to rising alpha diversity by reducing the dominance of early-life taxa such as *Bifidobacterium* and other early-life abundant taxa, including *Staphylococcus*, *Streptococcus*, *Veillonella*, and *Lactobacillus*. Together, these transitions reconfigure community structure and function, marking a critical shift toward a more diverse and adult-like gut microbiota [21,23].

The period of complementary feeding (approximately 6–24 months of age) is now recognized as a critical window for shaping both immediate and long-term health outcomes [20,24]. The World Health Organization (WHO) recommends exclusive breastfeeding for the first six months of life, followed by continued breastfeeding alongside complementary food up to two years of age or beyond [25]. In practice, the age of complete weaning varies widely depending on geographic, cultural, and socioeconomic factors. In high-income countries, weaning often begins and ends earlier than recommended; for example, only 26% of infants were still breastfed at 11 months old in a multi-site longitudinal study conducted in the United States, Finland, Germany, and Sweden [26]. In low- and middle-income countries, breastfeeding is typically continuing into toddlerhood, often until two years old or later. Notably, an extended breastfeeding period may maintain a breastmilk-oriented microbiota for a longer period, whereas early weaning could lead to a more rapid transition to an adult-like microbiota. To date, few studies have directly compared gut microbiota development in children aged 6–24 months who continue breastfeeding alongside complementary foods versus those weaned at similar ages, and existing evidence has been inconclusive [20,27,28,29]. It is also worth noting that both the timing of introducing solid food and the timing of breastfeeding cessation matter, as they could independently affect gut microbiota and have long-term effects. Nevertheless, Parkin et al. [30], reported that among predominantly breastfed infants, introduction of solids before 5 months (versus after 6 months) was associated with significantly higher alpha-diversity, primarily driven by a reduction in *Bifidobacterium*. In contrast, no such association was observed between the age of solid food introduction and microbial diversity in formula-fed infants, suggesting that breast milk, independent of complementary food and distinct from formula, plays a unique role in shaping gut microbiota during the complementary feeding period. However, this area is largely unexplored.

This study aimed to address the aforementioned knowledge gap by examining the influence of breastfeeding, rather than the timing or specific types of complementary food, on gut microbiota development during the complementary feeding period spanning from 6 to 24 months of age. We hypothesized that the gut microbiota composition continued to undergo age-dependent maturation during the complementary feeding period (6 to 24 months of age) in Peruvian Amazonian children, and that distinct microbial succession trajectories emerged between children who continued breastfeeding during this period and those who were fully weaned from breastfeeding. The study cohort resided in the Loreto Province of Peru, a region where the early introduction of complementary foods (often as early as two months of age) combined with prolonged breastfeeding to two years is common [31]. These feeding patterns offered a unique opportunity to investigate a wide range of breastfeeding durations during the first two years of life within a single population. This study enhances our understanding of how delayed weaning beyond infancy influences gut microbiota succession. Furthermore, it provides insights into the potential health implications of prolonged breastfeeding, thereby contributing to evidence-based recommendations regarding the optimal duration of breastfeeding and complementary feeding from a gut microbiota perspective.

## 2. Materials and Methods

### 2.1. Sample and Data Collection

This study utilized data from the Peruvian cohort of the Etiology, Risk Factors, and Interactions of Enteric Infections and Malnutrition and the Consequences for Child Health and Development (MAL-ED) project. MAL-ED is a multi-center, prospective, longitudinal observational study conducted across low-resource settings in eight developing countries, characterized by high rates of diarrheal disease and malnutrition [32]. At the time of initial analysis, the Peruvian site was the only MAL-ED cohort with publicly available epidemiological data and 16S rRNA gene sequencing data for fecal samples.

The trained field staff visited each participant’s home twice a week from birth to 24 months of age. During these visits, they collected daily reported data from mothers or caregivers on illness, treatments, and feeding practices using standardized forms [32,33,34,35,36]. Based on the bi-weekly feeding practice reports, the MAL-ED study tracked transitions in feeding practices and categorized them into distinct groups. The main categories of breastfeeding status in MAL-ED study include: exclusive breastfeeding (only breast milk was given, no other liquids or solids, except for medicines or vitamins), predominant breastfeeding (breast milk and water or water-based drinks, e.g., juice, tea, but no other milks, formula, solids, or semi-solids), partial breastfeeding (breast milk along with other milks, formula, semi-solid, or solid foods), and completed weaned (cessation of breastfeeding). The train field staff also collected biological samples during periodic visits. Non-diarrheal fecal samples were collected monthly for all children during the first year of life, and quarterly (every three months) in the second year of life.

### 2.2. Sample Processing

Fecal samples underwent DNA extraction, PCR amplification, and sequencing of the V4 hypervariable region of the 16S rRNA gene using Illumina MiSeq paired-end sequencing. Sequencing protocols and quality control measures have been described elsewhere [37]. Raw demultiplexed 16S rRNA sequence reads were obtained from the European Nucleotide Archive (ENA; accession number PRJEB28159), and associated epidemiological metadata were retrieved from the Clinical Epidemiology Database (ClinEpiDB).

The sequenced fecal samples were included in the study based on three criteria: (1) collected within the study age range: 6 months (6M, 170–190 days), 12 months (12M, 350–370 days), 18 months (18M, 544–552 days), and 24 months (24M, 721–730 days); (2) absence of diarrhea at the time of collection; (3) breastfeeding status within the MAL-ED categories of predominant breastfeeding, partial breastfeeding, or completed weaned. From the Peruvian cohort, we identified 778 non-diarrheal fecal samples from 265 infants and toddlers (123 females and 142 males).

We then assigned forward reads to samples and truncated them at 248 bp to remove low-quality base calls. Quality filtering and denoising were performed using the DADA2 algorithm [38] implemented through QIIME 2 (version 2024.10) with the q2-dada2 plugin, following established protocols [38,39,40]. DADA2 enhances data processing by reducing spurious sequences and minimizing residual error rates while preserving more biologically relevant amplicon sequence variants (ASVs) and retaining a greater portion of reads compared to traditional Operational Taxonomic Unit (OTU) clustering approaches.

Following quality control, we retained 702 samples. One sample from the 6M post-weaning group was excluded because it was the only sample in that category. As a result, we retained 701 high-quality fecal samples from 261 participants (119 females and 142 males) for analysis. A flowchart illustrating the reduction in sample count from 778 to 701 is shown in Supplementary Figure S1. Among the 261 participants, 44 contributed samples at one time point, 54 at two time points, 103 at three time points, and 60 at all four time points (Supplementary Table S1). To maximize data utilization, we employed a mixed-longitudinal approach by combining the available samples and conducting cross-sectional comparisons at each time point.

### 2.3. Study Groups

Qualified fecal samples were categorized into two groups: the complementary feeding (BF) group and the post-weaning (NBF) group. The BF group included children who were receiving both breast milk and complementary foods (liquid, semi-solid, and/or solid) at the time of sampling, corresponding to the “predominant” and “partial” breastfeeding classifications in the MAL-ED study. Among the 519 BF samples, 65 (12.5%) were from children who were predominantly breastfed, while the remaining samples represented partial breastfeeding. The NBF group consisted of children who had completed weaning, aligning with the “completely weaned” category in MAL-ED. Samples were further stratified by age into four time points: 6M, 12M, 18M, and 24M, as described in Section 2.1. Detailed sample composition and counts by age and feeding group are provided in Supplementary Table S2.

The Peruvian cohort resided in Loreto Province, a resource-limited region, with a high incidence of early childhood mortality and diarrheal disease [36]. In Loreto, breastfeeding is nearly universal, with 99% of infants being breastfed. Prolonged breastfeeding into the toddler years is common; 75% of children are partially breastfed beyond 17 months and older [36,41,42]. Complementary foods, including fish, local fruits, vegetables, and starchy crops such as yucca and banana, are often introduced as early as two months of age alongside continued breastfeeding [31]. The types and timing of complementary food introduction are largely influenced by seasonal variation and local resource availability. Previous reports have suggested that such diets may not consistently meet the WHO-recommended intake for essential micronutrients [31,36]. Acute undernutrition is relatively uncommon in the Peruvian MAL-ED cohort. However, stunting remains highly prevalent throughout Peru; at 24 months of age, approximately 36% of children were classified as stunted, defined by a length-for-age Z-score below −2 [43].

### 2.4. Diversity and Taxonomy Analyses

We placed ASVs into the Greengenes2 reference database [44] using the SATé-enabled phylogenetic placement (SEPP) algorithm via the QIIME 2 plugin. Detailed descriptions of the Greengenes2 reference framework, SEPP insertion pipeline, and classifier parameters are available in prior studies [39,44,45,46,47,48]. In line with the study’s focus on dominant microbial taxa, only high-abundance ASVs were retained for downstream analysis.

Alpha diversity, reflecting within-sample diversity, was evaluated using two metrics. Faith’s Phylogenetic Diversity is related to species richness; it measures the total branch length of the phylogenetic tree connecting all taxa observed in a sample, thereby incorporating phylogenetic relationships among taxa and providing a phylogenetically informed assessment of diversity [39,49,50,51,52]. Pielou’s Evenness assesses the uniformity of species abundance distributions within the sample [39,53]. We calculated these measures for each sample and compared them across age groups and between the BF vs. NBF groups. We used Kruskal–Wallis tests to evaluate overall differences in alpha diversity across multiple groups, with Dunn’s test for pairwise comparisons [54,55,56,57]. We then used Benjamini–Hochberg for false discovery rate (FDR) correction [39,57], with *q* < 0.05 indicating statistical significance.

Beta diversity, which reflects between-sample diversity, assesses the differences in microbial community composition. Community dissimilarities were quantified using the Weighted UniFrac distance, which incorporates both phylogenetic relationships and relative abundances of taxa [58]. We visualized clustering patterns by age and breastfeeding status using principal coordinates analysis (PCoA) [39,58,59,60,61,62]. To test for statistically significant differences in community composition across groups (by age or breastfeeding status), we applied permutational multivariate analysis of variance (PERMANOVA) to the UniFrac distance matrices with 999 permutations [63]. To verify that observed PERMANOVA significance was not driven by differences in group dispersion, we performed a permutational analysis of multivariate dispersions (PERMDISP) for each comparison. A significant PERMANOVA (*p* < 0.05) result accompanied by a non-significant PERMDISP result (*p* > 0.05) was interpreted as evidence of true compositional differences rather than dispersion-related artifacts [39,64].

Taxonomic classification of ASVs was conducted using the q2-feature-classifier plugin in QIIME 2 [59], with a naïve Bayes classifier trained on the Greengenes2 reference sequence database [44]. For each sample, relative abundance was calculated for each taxon as the proportion of total sequences assigned to that taxon [65]. We identified the most abundant phyla and genera across the dataset and compared their relative abundances across age groups and between the BF and NBF groups. Using rarefied data, we applied non-parametric Kruskal–Wallis tests to assess overall differences in the relative abundance of each major taxon across age groups within the BF and NBF, respectively. Significant results were followed by pairwise comparisons using Dunn’s test with Benjamini–Hochberg FDR correction [39,54,55,56,57]. Additionally, we compared the relative abundances of key taxa between BF and NBF groups at each time point (12M, 18M, and 24M) to identify taxa significantly associated with breastfeeding status. Statistical significance was set at *q* < 0.05 after correction for multiple testing.

We used QIIME 2 (version 2024.10), R (version 4.2.3), and RStudio (version 2025.05.1+496) to visualize data and perform statistical analysis. Data supporting the findings of this study are available from the authors upon request.

### 2.5. Metadata Analysis

We analyzed metadata from the study cohort to evaluate and control for potential confounding factors between the BF and NBF groups at 12M, 18M, and 24M of age. Supplementary Tables S3–S8 provide the complete set of the relevant metadata variables in the following categories: Demographics, breastfeeding status and ages, complementary feeding status and ages, vaccination, diarrhea, and illnesses. We assessed the normality of continuous variables using the Shapiro–Wilk test [66], and tested for homogeneity of variance using the F-test [67]. For variables that met both assumptions of normality and equal variance, we applied Student’s *t*-test to evaluate group differences [68]. When these assumptions were violated, we used the non-parametric Kruskal–Wallis test [54]. Categorical variables were analyzed using Fisher’s exact test, which is appropriate for small sample sizes and unequal distributions [69,70]. For non-normally distributed data, we reported the median and interquartile range (IQR; Q1–Q3) as measures of central tendency and dispersion. All statistical analyses were conducted in R (version 4.2.3).

### 2.6. Multivariable Association Analysis

We used the Multivariable Association with Linear Models 2 (MaAsLin2) R package (version 1.22.0) [71] to assess the relationships between gut microbiota composition and key variables of interest, specifically child age and breastfeeding status. To control for potential confounding, we included in the model a set of relevant covariates derived from available metadata, including age at cessation of exclusive breastfeeding (in days), whether colostrum was fed, breastfeeding initiation within the first 24 h, prelacteal feeding, age at first exposure to animal milk or formula, age at first introduction of clear liquids, and age at first introduction of solid foods.

MaAsLin2 was run using default parameters unless otherwise specified. Microbial features, specifically, alpha diversity measures and taxonomic relative abundances, were treated as dependent variables. Fixed effects included the primary variables of interest as well as all specified covariates. Alpha diversity metrics (Faith’s phylogenetic diversity and Pielou’s evenness) were analyzed without transformation or normalization, as they were already computed as continuous summary statistics. Statistical significance was defined as an FDR–adjusted *q* < 0.05, using the Benjamini–Hochberg procedure to correct for multiple testing. All results are reported with corresponding effect sizes, standard errors, *p* values, and *q* values.

## 3. Results

Following sequence processing and quality control, data from 261 children (119 females and 142 males) were included, comprising a total of 701 stool samples. Among these, 519 samples were from the complementary feeding with breast milk (BF) group and 182 from the post-weaning (NBF) group. The age- and feeding-status distribution of the samples was as follows: 213 samples at 6M (all from the BF group, as nearly all children in this cohort were still breastfeeding at this age); 187 samples at 12M (180 BF, 7 NBF); 180 samples at 18M (103 BF, 77 NBF); and 121 samples at 24M (23 BF, 98 NBF). This distribution reflects the progressive increase in weaning over time. Additional details are provided in Supplementary Tables S1 and S2.

### 3.1. Alpha Diversity

#### 3.1.1. Species Richness Across Ages and Between BF and NBF Groups

**Figure 1:**
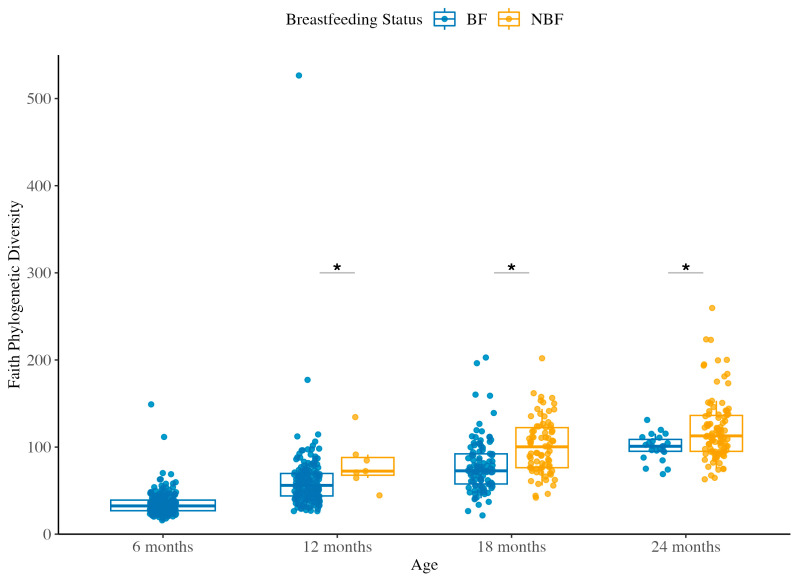
Species richness across ages between BF and NBF groups. Faith’s Phylogenetic Diversity measurement was used to assess species richness in the gut microbiota of Peruvian children at 6, 12, 18, and 24 months of age, stratified by breastfeeding status: BF (breastfeeding with complementary food) and NBF (post-weaning). Each dot represents an individual sample, and boxplots show the interquartile range (IQR) with the median as the horizontal line and whiskers extending to 1.5 × IQR. Alpha diversity values were calculated after rarefaction to 21,059 sequences per sample, retaining 64.71% of features in 90.23% of samples. Statistical comparisons between BF and NBF groups at each time point were performed using the Kruskal–Wallis test followed by Dunn’s post hoc test, with false discovery rate (FDR) correction using the Benjamini–Hochberg method. Asterisks (*) indicate statistically significant differences (*q* < 0.05) between BF and NBF groups. Detailed statistical outcomes are presented in Supplementary Table S9A,B.

Species richness was assessed using Faith’s Phylogenetic Diversity. This metric accounts for the total branch length of the phylogenetic tree covered by the community in a sample, thus reflecting the diversity of lineages present. In the BF group comprising children who received breast milk and complementary food, species richness increased significantly with age (Kruskal–Wallis *p* < 2.2 × 10^−16^), indicating a clear maturation pattern as children grew older and their diets diversified (Figure 1). All pairwise comparisons between age groups in BF showed significant increases in richness (*q* < 0.05 after FDR correction).

The NBF group, comprising children who had stopped breastfeeding, also exhibited an overall age-related increase in species richness (Kruskal–Wallis *p* = 0.0001). However, the pattern in NBF was less uniform than in BF. Pairwise comparisons revealed that significant gains in richness were observed mainly between more widely spaced time points in late second year (*q* = 0.0037 for 12M vs. 24M, *q* = 0.0017 for 18M vs. 24M), whereas the difference between 12M and 18M in NBF was not significant (*q* = 0.0793). The results suggest that in the absence of ongoing breastfeeding, the accumulation of new microbial lineages in the gut may occur in a more stepwise or variable manner, with less incremental change between the 12M and 18M intervals, but a larger jump by 24M.

When comparing species richness between BF and NBF groups at the same age, we found that the NBF group consistently exhibited higher species richness than the BF group at each matched time point (Figure 1 and Supplementary Table S9B). In practical terms, by 12M of age and beyond, children who had been weaned harbored more phylogenetically diverse gut communities than those who were still receiving breast milk. This finding suggests that breastfeeding, while supporting a specialized microbiota enriched in HMO-associated microbes (e.g., *Bifidobacterium*), may transiently limit the breadth of microbial lineages, whereas early weaning allows a broader range of microbes to establish by the second year of life. In summary, the richness of gut microbial communities increased with age in both groups. However, children who received breast milk during the complementary feeding period followed a smoother, incremental diversification and remained remarkably less diverse than post-weaning children between 12M and 24M old, implying that the cessation of breastfeeding affected the microbiota succession trajectory in addition to complementary food.

#### 3.1.2. Species Evenness Across Ages and Between BF and NBF Groups

**Figure 2:**
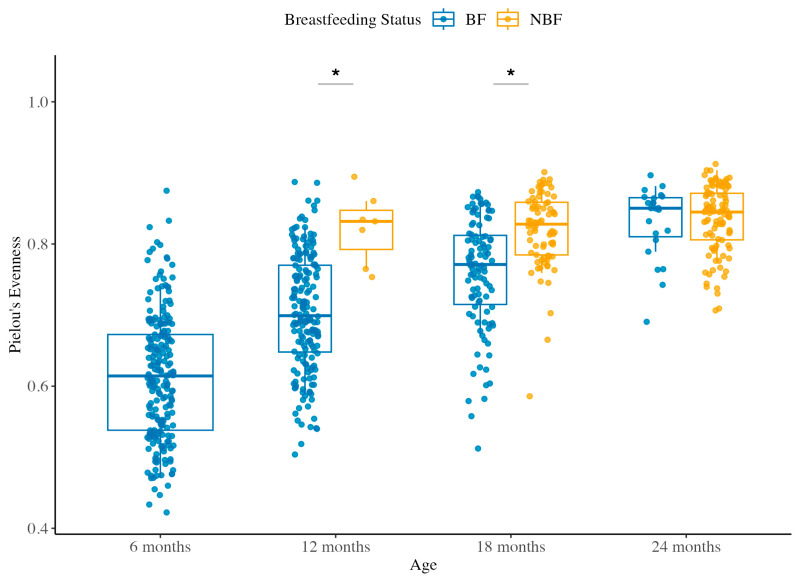
Species evenness across ages between BF and NBF groups. Species evenness was analyzed in the gut microbiota of Peruvian children at 6, 12, 18, and 24 months of age, stratified by breastfeeding status: BF (breastfeeding with complementary food) and NBF (post-weaning). Each dot represents an individual sample, and boxplots show the interquartile range (IQR) with the median as the horizontal line and whiskers extending to 1.5× IQR. Alpha diversity values were calculated after rarefaction to 21,059 sequences per sample, retaining 64.71% of features in 90.23% of samples. Statistical comparisons between BF and NBF groups at each time point were performed using the Kruskal–Wallis test followed by Dunn’s post hoc test, with false FDR correction using the Benjamini–Hochberg method. Asterisks (*) indicate statistically significant differences (*q* < 0.05) between groups. Detailed statistical outcomes are presented in Supplementary Table S10A,B.

We next assessed Pielou’s evenness, which measures the evenness of distribution of individual organisms among the species present in a community. As shown in Figure 2, the value for evenness ranges from 0 to 1, with higher values indicating a more even community. In the complementary feeding BF group, species evenness showed a significant increasing trend with age (Kruskal–Wallis *p* < 2.2 × 10^−16^). Pairwise comparisons confirmed that the evenness in BF rose steadily from 6M to 24M (all *q* < 5.46 × 10^−3^), suggesting that as breastfed children grow and consume more varied diets, their gut communities transition from being dominated by a few taxa (e.g., *Bifidobacterium* in early infancy) to a more even distribution of abundances by 24M of age.

In contrast, the post-weaning NBF group did not exhibit a significant overall change in evenness across 12M, 18M, and 24M (Kruskal–Wallis *p* = 0.1551, Figure 2). The evenness values in NBF were relatively high as early as 12M without showing any trending changes thereafter, implying that once children are weaned, their microbiota may quickly attain a certain level of evenness, possibly due to early colonization by a variety of microbes associated with the introduction of complementary food, and further aging from 12M to 24M does not markedly alter the general abundance distribution.

Between the complementary feeding BF and post-weaning NBF groups, we observed that at 12M and 18M, the BF had significantly lower evenness than the NBF (*p* = 0.0004 and *p* = 1.54 × 10^−8^, respectively). This aligns with the notion that continued breastfeeding maintains a dominance of a few key taxa (lower evenness), whereas weaned children at these ages already have more equilibrated communities. By 24M, the difference in evenness between BF and NBF groups disappeared (*p* = 0.8949), with BF children’s gut microbial evenness catching up to a level comparable to their weaned counterparts (Figure 2). This convergence by the age of two years suggests that the influence of breastfeeding on community evenness is pronounced in the early toddlerhood but diminishes as children approach the end of the second year, likely due to the increasing impact of a shared diet and environment overriding early-life feeding mode effects.

In summary, breastfeeding during the complementary feeding period was associated with a less even distribution of gut microbes in the first 1–1.5 years, while early-weaned children had a more even community from the outset of complementary feeding. By the age of two years, both groups had attained a similar level of species evenness. These alpha diversity patterns (richness and evenness) indicate that prolonged breastfeeding delays certain aspects of microbiota diversification and evenness, although both groups ultimately trend toward a mature state by age two.

### 3.2. Beta Diversity Across Ages and Between BF and NBF

**Figure 3:**
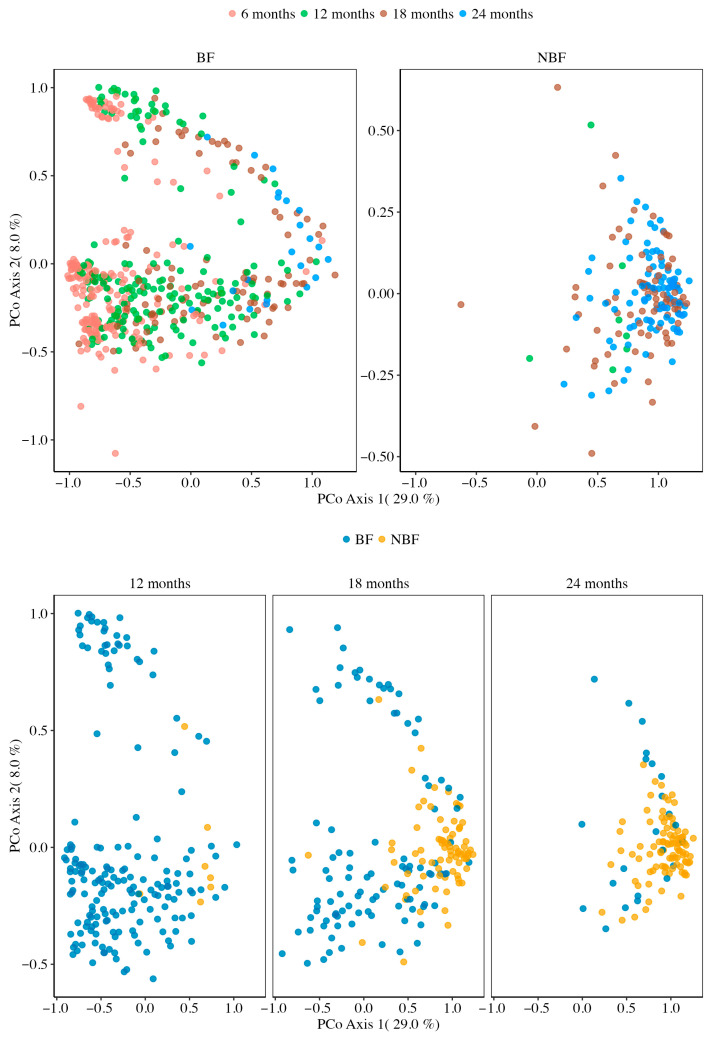
Beta diversity across age groups and between BF and NBF. Principal Coordinate Analysis (PCoA) was performed using the Weighted UniFrac distance, a phylogenetic beta-diversity metric that quantifies dissimilarity between microbial communities by incorporating both the relative abundances of taxa and their evolutionary relationships in a phylogenetic tree. Each dot represented a single sample. Samples from the 6, 12, 18, and 24 months of age are shown for the BF. Samples from the 12, 18, and 24 months of age are shown for the NBF groups. The spatial proximity between points indicates similarity in microbial community structure; greater distances reflect higher dissimilarity. PCoA axis 1 and axis 2 represent the first and second principal coordinates, explaining the largest amount of feature variation. PERMANOVA, followed by PERMANOVA Pairwise comparison, was used to evaluate the statistical differences between age groups. The PERMDISP test was conducted to ensure observed differences were not due to heterogeneity of dispersion. Multiple testing was corrected using the Benjamini–Hochberg procedure, and *q*-values are reported to control the false discovery rate. Detailed statistical results are provided in Supplementary Table S11A,B.

Beta diversity analysis provided insights into differences in overall microbial community composition between samples, considering phylogenetic relatedness of microbes. Within the complementary feeding BF group, we observed distinct clustering of samples according to age along the principal coordinate axes (Figure 3, BF panel, overall *p* = 0.001), suggesting a significant overall effect of age on gut microbiota composition. All the pairwise PERMANOVA comparisons between age groups in BF were significant after FDR correction (all *q* = 0.0012 except for *q* = 0.0110 for 18M vs. 24M), suggesting that each jump in age contributed to a measurable shift in the microbial community composition.

To discern whether these age-related differences were due to changes in community composition per se or due to differences in within-group variability (dispersion), we performed PERMDISP. The overall PERMDISP across the four BF age groups was significantly different (*p* = 0.0001), indicating that the dispersion of samples differed among age groups. Pairwise PERMDISP tests in BF samples showed that the dispersion at 6M was significantly higher compared to 12M and 18M (*p* < 0.05 for 6M vs. 12M and 6M vs. 18M), whereas there were no significant dispersion differences for the comparisons that did not involve 6M (i.e., 12M vs. 18M, 12M vs. 24M, and 18M vs. 24M all had *p* > 0.05), indicating that part of the separation observed between 6M and later ages on the PCoA is attributable to the 6M samples being more heterogeneous. For age comparisons beyond 6M, the differences in clustering can be more confidently attributed to true shifts in community composition, since dispersions were similar. These findings suggest that the gut microbiotas of children who continued receiving breast milk experienced the most significant community restructuring between 12M and 24M old, alongside a reduction in variability as the microbiota matured. The high dispersion observed in the BF samples at 6M align with the well documented phenomenon of high inter-individual variability in the gut microbiota of breastfed infants (children of 12M or younger), mainly due to initial colonization after birth, mother-specific breast milk composition and microbiota, and household environment [72,73,74,75].

In the post-weaning NBF group, an overall age effect on beta diversity was also detected, but it was less pronounced than in the BF, as shown in the NBF panel of Figure 3 (*p* = 0.022). Further pairwise PERMANOVA revealed a more limited pattern, with only the 12M vs. 24M comparison being significant (*q* = 0.039), suggesting that in weaned children, the gut microbial community structure at 12M and 18M was relatively similar, with a more noticeable shift only by 24M. Notably, PERMDISP for NBF age groups was not significant (overall *p* = 0.3693), indicating that variability within each age group was comparable. Therefore, the significant separation between the 12M and 24M NBF samples can be attributed to genuine compositional change over that period, rather than inter-individual heterogeneity. In essence, the microbiota of post-weaning children underwent age-related changes, but these changes were subtler and mostly detectable when comparing the start and end of the second year.

When comparing the BF and NBF groups at each age point (12M, 18M, and 24M), we observed distinct clustering of BF and NBF samples (Figure 3, lower panel). PERMANOVA tests confirmed a statistically significant difference in community structure between the BF and NBF at all three age points (*p* ≤ 0.024). Further PERMDISP tests confirmed the absence of significant dispersion between the BF and NBF groups at each respective age (*p* ≥ 0.1045), indicating that the compositional differences are not confounded by unequal within-group variability.

In summary, these findings demonstrate that (1) prolonged breastfeeding over infancy imprint on the gut microbiota in a way that remains detectable up to at least 24M; (2) gut microbiota presents high dispersion in the first year of life and becomes more homogeneous between individuals of the same age and under similar feeding patterns in toddler years.

### 3.3. Taxonomy

#### 3.3.1. Microbial Composition at Phylum Level

**Figure 4:**
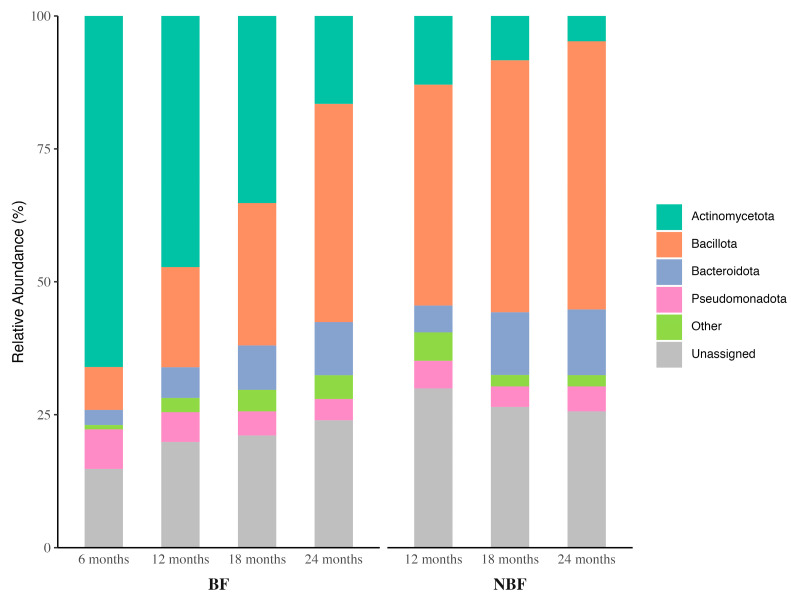
Microbial composition at the Phylum level. The relative abundance of the four most abundant phyla across ages and between BF and NBF groups was depicted by the stacked bars. Kruskal–Wallis and Kruskal–Wallis pairwise tests were used to determine the significance between ages and between the BF and NBF groups. Statistical analysis results are provided in Supplementary Table S12A (age effect) and S12B (BF vs. NBF comparisons).

Next, we conducted a relative abundance analysis to identify the dominant microbial taxa in the complementary feeding BF group and the post-weaning NBF group. The results demonstrated that four major bacterial phyla dominated the gut microbiota in this cohort: Actinomycetota (formerly Actinobacteria; largely comprising *Bifidobacterium* in early childhood), Bacillota (formerly Firmicutes; including Clostridia and Bacilli classes), Bacteroidota (formerly Bacteroidetes), and Pseudomonadota (formerly Proteobacteria; including *Escherichia* and other Gammaproteobacteria). Together, these phyla accounted for the majority of sequences in all samples, though their relative proportions shifted with age and feeding status (Figure 4 and Supplementary Table S12A,B for statistical analysis results).

Within the BF group, we observed significant changes in the relative abundance at the phylum level across ages (Kruskal–Wallis *p* < 2.2 × 10^−16^). Pairwise comparisons revealed distinct temporal patterns for each phylum as BF children grew older. Actinomycetota, the most abundant phylum in infant gut microbiota, exhibited a remarkable continuous decline from 6M (66.0%) to 24M (16.7%), with statistical significance across all pairwise age comparisons (*p* ≤ 1.154 × 10^−5^). The decreasing trend was also observed for Pseudomonadota, which was noticeable in the 6M vs. 12M and 6M vs. 18M comparisons, but not significant in the second-year comparisons, reflecting an early shift and quick stabilization at the beginning of the second year. In contrast to the declining trend of Actinomycetota and Pseudomonadota, Bacillota proliferated from 8.1% to 41.0% (*p* ≤ 0.0002 for all comparisons) and Bacteroidota from 2.8% to 10% (*p* ≤ 0.0062 except for 18M vs. 24M) in the first two years, suggesting that Bacillota and Bacteroidota become established gradually when children mature and progress to adult-like gut microbiota despite receiving breast milk alongside complementary food. Notably, Bacteroidota seemed to achieve stabilization around 18M, earlier than Bacillota.

In the post-weaning NBF group, the overall relative abundance of microbial phyla is also significantly changed across ages (Kruskal–Wallis *p* < 2.2 × 10^−16^), with the same four phyla being dominant; however, their temporal patterns differ from those of the BF. At 12M, Actinomycetota had already dropped to a much lower abundance level in the NBF (12.9%) compared to the BF (47.3%), then a slower further decrease to 4.8% at 24M, which is consistent with the decline of *Bifidobacterium*, a well-known predominant genus of Actinomycetota reflecting breastmilk-supported colonization. The less profound changes in Actinomycetota in the NBF group align well with the post-weaning status throughout the second year. In contrast to Actinomycetota, the relative abundance of Bacteroidota in the NBF group increased significantly when comparing 12M to 24M (*p* = 0.0362), in agreement with the increasing microbial diversification during gut microbiota maturation. In addition, different from the BF group, no significant changes were detected in Bacillota or Pseudomonadota across any pairwise comparisons between age groups in the NBF, implying that those two phyla established dominance and stabilized quickly after introduction of complementary food when breast milk was not present, remaining relatively constant with aging in weaned children.

We further compared each of the four major phyla between the BF and NBF at the same time point to gain a better understanding of how breast milk affects the microbial relative abundance in the second year of life. In this study cohort, Actinomycetota was the most abundant phylum in BF children until 18M and consistently remained at a much higher level than in the NBF children at all age points (*p* ≤ 0.0002), supporting the strong role of breast milk in sustaining *Bifidobacterium* throughout the complementary feeding period, including into the toddler year. In contrast, Bacillota had become the most dominant phylum in NBF children by 12M, if not earlier (6M post-weaning data are unavailable), and maintained a persistently higher abundance than in BF children at all time points (*p* ≤ 0.0127). Bacteroidota, another rising phylum during gut maturation, was the third most abundant phylum in the BF and NBF children. Its relative abundance was comparable at 12M (*p* = 0.8419) between the BF and NBF children, higher in the NBF than BF at 18M (*p* = 0.0380), and eventually converging by 24M (*p* = 0.6676). Apart from the other three dominant phyla, the Pseudomonadota did not emerge as a distinguishing phylum between the BF and NBF groups, as all comparisons between the two groups were statistically insignificant, implying its abundance is less influenced by feeding practices in this cohort.

#### 3.3.2. Microbial Composition at Genus Level

**Figure 5:**
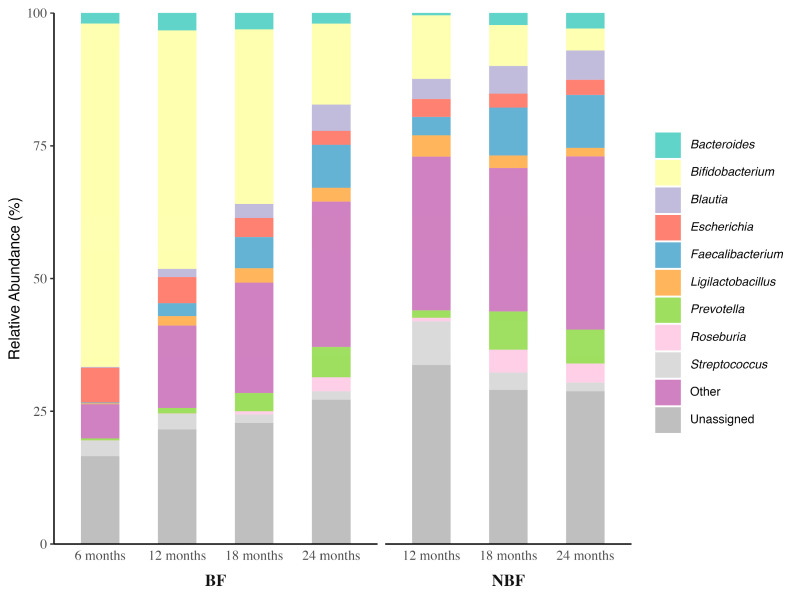
Microbial composition at the Genus level. The relative abundance of the most abundant genera across ages and between BF and NBF groups was depicted by the stacked bars. Kruskal–Wallis and the following pairwise tests were used to determine the significance between ages and between the BF and NBF groups. Statistical analysis results are provided in Supplementary Table S13A (age effect) and S13B (BF vs. NBF comparisons).

We also analyzed the relative abundance at the genus level. The identified top nine most abundant genera across all align seemingly with the aforementioned dominant phyla, including *Bifidobacterium* in the phylum Actinomycetota, five genera *(Blautia*, *Faecalibacterium*, *Ligilactobacillus*, *Roseburia*, and *Streptococcus)* in the phylum Bacillota, two genera *(Bacteroides* and *Prevotella)* in the phylum Bacteroidota, and *Escherichia* in the phylum Pseudomonadota. Figure 5 illustrates the relative abundance trends, and Supplementary Table S13A,B provide the detailed statistics.

Within the BF group, the overall genus composition changed significantly with age (*p* < 2.2 × 10^−16^), mirroring the phylum-level shifts. The specific temporal dynamics for each genus are as follows. As breastfed children grew older, *Bifidobacterium*, a characteristic and most abundant genus of infant gut microbiota [76,77], showed significant decreases in abundance across all pairwise age comparisons (*p* ≤ 1.677 × 10^−5^). Conversely, genera typically associated with a more diversified diet rich in complex carbohydrates and proteins [78,79], such as *Blautia*, *Faecalibacterium*, and *Roseburia*, exhibited continuous and significant increases in abundance across nearly all pairwise age comparisons (*p* ≤ 0.0064). In a consistent manner, *Prevotella*, another key bacterial genus in the maturing gut microbiota, especially in response to high-fiber plant-based diets [80,81], also rose continuously (all *p* ≤ 2.356 × 10^−6^, except for *p* = 0.0646 for 18M vs. 24M). *Bacteroides*, belonging to the same phylum as *Prevotella*, also proliferated significantly in the first year but became stabilized around 12M, earlier than *Prevotella*. *Escherichia* and *Streptococcus*, which are often abundant in early infancy, showed significant decreases in abundance from 6M to 18M and stabilized in the late second year.

For the NBF group, the overall microbial composition, in terms of the genus-level relative abundance, also demonstrated significant differences across the age groups (Kruskal–Wallis *p* < 2.2 × 10^−16^). However, unlike the BF group, which showed significant temporal changes in each top genus, the NBF either achieved a steady state earlier or displayed attenuated changes, as statistical significance was often detected when comparing 12M vs. 24M. Regarding the genera *Bifidobacterium*, *Faecalibacterium*, *Roseburia*, *Streptococcus*, *Bacteroides*, and *Prevotella*, the NBF group exhibited similar directional trends to the BF group over the same age range. No statistically significant age-related changes were detected for *Blautia* and *Escherichia* in the NBF group, despite an apparent increase in *Blautia* and decrease in *Escherichia* in the BF group. This pattern suggests that these genera may establish stable populations shortly after the weaning period. The relative abundance of *Ligilactobacillus* significantly increased in the BF group but declined in the NBF group. *Ligilactobacillus* was formerly part of the broader *Lactobacillus* genus, a characteristic genus in the gut microbiota of young, breastfed infants [82] like *Bifidobacterium*. While *Bifidobacterium* abundance declined in BF children during the complementary feeding period, the concurrent increase in *Ligilactobacillus* may speculatively reflect its adaptation to the changes in mother’s milk microbiota and/or milk components during toddler years [83,84,85]. This interpretation is further supported by the decreasing trend of *Ligilactobacillus* observed in the NBF group.

When comparing each genus between BF and NBF at a specific age time point, it became clear that the BF samples were more abundant in breast-milk-orientated *Bifidobacterium* (often 3–4-fold higher) and *Ligilactobacillus*. Regarding the food-related taxa, the comparison results varied. For *Blautia*, *Faecalibacterium*, *Prevotella*, and *Streptococcus*, the BF groups showed significantly lower abundance than the NBF at 12M and 18M, but became comparable by 24M. *Roseburia* was persistently lower in BF than the NBF, while *Bacteroides* and *Escherichia* remained the same between the two groups throughout the second year of life.

Overall, our phylum- and genus-level analyses demonstrate that prolonged breastfeeding into the second year of life, during the complementary feeding period, consistently and significantly influences the abundance of key genera. These include genera associated with breast milk digestion (*Bifidobacterium* and *Ligilactobacillus*) as well as those linked to dietary diversification (*Blautia*, *Faecalibacterium*, *Prevotella*, *Roseburia*, and *Streptococcus*). These bacteria play essential roles in gut microbiota maturation, immune modulation, and nutrient metabolism, and have been associated with child growth and overall health [24,77,86,87].

### 3.4. Comparison of Potential Confounding Factors

Recognizing that factors beyond feeding practices may influence gut microbiota composition, we examined available metadata to identify potential confounders differing between the BF and NBF groups. Demographic and health-related variables, including child sex, birth weight, vaccination status, and indicators of illness or diarrhea, were compared between the two groups at 12M, 18M, and 24M of age. As detailed in Supplementary Tables S3, S6–S8, no significant differences were detected in any of these variables across time points. These findings suggest a low likelihood that the observed microbiota differences arose from underlying population stratification, supporting the interpretation that feeding practices were the primary drivers of the microbial variation observed.

As expected by the study design, the BF and NBF groups differed significantly in breastfeeding-related variables (Supplementary Table S4). BF children were still receiving breast milk at the time of stool sample collection, whereas all NBF children had already been weaned for over 7 days. The median age at complete weaning for the NBF is approximately 9.3 months (279 days) for the 12M group, 15.8 months (474 days) for the 18M group, and 18.4 months (551.5 days) for the 24M group. Accordingly, the BF had significantly longer breastfeeding durations, approximately 80 to 180 days longer, depending on the age group. These expected differences validate the group classifications and underscore that breastfeeding duration and intensity were among the primary exposures distinguishing the gut microbiota profiles.

Additionally, we examined complementary feeding practices for potential differences (Supplementary Table S5). Important confounding factors, including the timing of dietary introduction of formula, animal milk, and solid foods, did not show significant differences. Interestingly, we found that the timing of introducing the first clear liquid (water or tea) was significantly earlier in NBF children at 12M and 18M; this may reflect that those mothers who weaned earlier introduced other fluids sooner.

In summary, our analysis of the metadata did not reveal any obvious confounders that could fully explain the differences in microbiota between the complementary feeding BF group and post-weaning NBF group. There were no systematic biases in health or vaccination between the groups, indicating that the persistent microbiota differences were most likely driven by feeding mode (breast milk exposure) and its associated nutritional factors. The earlier introduction of certain foods or liquids in the post-weaning group is an inherent part of the weaning process and may mechanistically contribute to the detected microbial features in this study. These nuances are taken into account in the discussion of our findings.

### 3.5. Effect Size of Age and Breast Milk Exposure

We performed multivariable association analysis with the MaAsLin2 model to estimate the effect size of age and breast milk exposure during the complementary feeding period on microbial features, with control of key covariates related to feeding mode, including colostrum intake, timing of breastfeeding initiation, and age at introduction of animal milk, clear liquids, and solid foods.

We found that age presents strong and consistent effects on nearly all tested microbial features. Child age is positively associated with species richness measured by Faith Phylogenetic Diversity (coef = 23.55, *q* = 7.16 × 10^−45^) and species evenness measured by Pielou’s Evenness (coef = 0.072, *q* = 2.68 × 10^−60^). Increasing Age has also been found to be associated with more abundance of *Faecalibacterium* (coef = 3.51, *q* = 2.17 × 10^−76^), *Blautia* (coef = 2.97, *q* = 5.45 × 10^−70^), *Roseburia* (coef = 1.65, *q* = 2.11 × 10^−32^), *Prevotella* (coef = 2.50, *q* = 4.29 × 10^−32^), *Ligitactobacillus* (coef = 2.33, *q* = 1.14 × 10^−26^), and *Bacteriodes* (coef = 1.77, *q* = 9.94 × 10^−19^), and negatively affected the abundance of *Bifidobacterium* (coef = −0.74, *q* = 4.21 × 10^−14^), *Streptococcus* (coef = −0.88, *q* = 1.72 × 10^−11^), and *Escherichia* (coef = −0.80, *q* = 1.35e × 10^−8^).

Breastfeeding status also showed significantly independent associations with microbiota composition, after adjusting for age and early-life covariates. Completion of weaning (NBF) had a substantial effect on species richness (coef = 20.07, *q* = 9.84 × 10^−8^) and a moderate effect on species evenness (coef = 0.037, *q* = 1.7 × 10^−4^), supporting that the presence of breastmilk during the second year of life slows down the diversification of gut microbiota. Among the identified top nine most abundant genera, NBF was found to be negatively associated with *Bifidobacterium* (coef = −3.60, *q* = 1.32 × 10^−51^) and *Ligilactobacillus* (coef = −2.55, *q* = 5.32 × 10^−7^) and positively associated with *Roseburia* (coef = 3.67, *q* = 3.25 × 10^−31^).

Except that the age of introduction of first animal milk or solid food had a mild effect on the relative abundance of *Faecalibacterium* (coef = −0.505, *q* = 0.03), we did not observe any association between any tested microbial features and the available factors related to feeding practice as listed in the Supplementary Tables S4 and S5. It is worth noting that the timing of introducing the first clear liquid (water or tea) was significantly earlier in NBF children at 12M and 18M; but it was not detected to be associated with any tested microbial features.

## 4. Discussion

Our study focuses on the impact of prolonged breastfeeding alongside complementary food, rather than exclusive breastfeeding, on the microbiota succession in children aged 6–24 months, covering late infancy and the first year of toddlerhood. We directly compared the children who continued receiving breast milk during the complementary feeding period (BF group) versus those who were fully weaned (NBF group) at similar ages. Previous evidence in this specific comparison has been limited. The unique feeding patterns observed in the Peruvian cohort, characterized by the early introduction of complementary food, often as early as two months of age, combined with prolonged breastfeeding extending into the toddler years, offered a great opportunity to investigate a wide range of breastfeeding durations within a single population. This design directly addresses a recognized gap in the literature, moving beyond the well-studied exclusive breastfeeding phase to focus on the less characterized yet crucial complementary feeding period, where feeding practices are highly variable globally and culturally. This approach offers a more nuanced understanding of how prolonged breast milk exposure, rather than its initial presence or complete absence, influences the gut microbiota. The findings provide valuable insights into the potential health implications of prolonged breastfeeding and contribute to evidence-based recommendations regarding optimal breastfeeding duration and complementary feeding from a gut microbiota perspective.

### 4.1. Summary of Study Findings About Impact of Prolonged Breastmilk on Gut Microbiota

The study found a clear and steady age-related increase in alpha diversity, measured by both species richness and evenness, across 6 to 24 months in the BF group. In contrast, the NBF group exhibited consistently higher species richness throughout the second year, but with less uniform changes over time. Evenness in the NBF group was already high by 12 months and remained stable, whereas in the BF group, evenness was significantly lower at 12 and 18 months but gradually increased to levels comparable to the NBF group by 24 months. The age-related increase in alpha diversity observed in our study is highly aligned with the well-documented microbiota maturation patterns in early childhood. However, the significantly delayed but steadier diversification in the BF group suggests that the presence of breast milk plays a profound role in shaping gut microbiota in the second year of life.

The beta diversity analysis provided further insights into the differences in overall microbial community composition between samples. The BF group exhibited distinct age-related clustering and compositional shifts across 12, 18, and 24 months. In contrast, NBF children showed fewer changes, with significant shifts primarily occurring when comparing the 24-month sample with the 12-month sample. At each age, the BF and NBF samples formed separate clusters, confirming divergent microbiota trajectories driven by feeding practices. This evidence further supports our hypothesis, suggesting that the presence of breast milk not only slows the pace of microbiota diversification but also steers its composition along a unique path in toddler years.

The relative abundance analysis at the phylum and genus levels provided more detailed information about the microbial community composition. The BF group retained higher Actinomycetota and lower Bacillota through 18 months, showing a delayed transition to an adult-like microbiota. In contrast, NBF children shifted earlier to Bacillota dominance. At the genus level, BF children consistently had a higher level of human-milk-orientated *Bifidobacterium* than NBF, despite a general downward trend. On the other hand, NBF children showed earlier and higher levels of genera associated with solid food digestion, such as *Blautia*, *Faecalibacterium*, *Roseburia*, *Prevotella*, and *Streptococcus*. *Bacteroides* and *Escherichia* levels were similar across groups. The divergent trajectories of *Ligilactobacillus*, rising in BF while falling in NBF, suggest its extended role in milk metabolism. These patterns reflect the unique impact of breast milk in prolonging the infant-type microbiota and modulating the pace of phylum- and genus-level shifts during microbiota maturation.

Our findings strongly suggest that breastfeeding, while supporting a specialized gut microbiota, transiently limits the breadth of microbial lineages and maintains lower evenness, whereas early weaning allows a broader range of microbes to establish more rapidly. This observation provides clear empirical evidence for a buffering effect of prolonged breastfeeding on microbiota diversification. Instead of simply delaying maturation, it appears to guide it along a distinct, more controlled trajectory, allowing for a gradual integration of new taxa rather than the abrupt shift often seen in early weaned children. These findings also support that prolonged breastfeeding modulates the gut maturation trajectory independently, in addition to the effects of introducing complementary foods.

### 4.2. Impact of Human Milk on Infant and Toddler Gut Microbiota

Extensive research over the past few decades has substantially enhanced our understanding of how breastfeeding influences the gut microbiota, particularly by comparing the fecal bacterial profiles of breastfed and formula-fed infants within the first year of life. Comprehensive reviews by others [17,20,88] have identified key distinctions, highlighting that breastfed infants typically exhibit lower bacterial diversity, whereas formula-fed infants often have microbiota profiles that more closely resemble mature, adult-like communities, as indicated by microbiota age or microbiota-for-age Z-scores [17,26]. This distinct composition in breastfed infants primarily arises from the elevated abundance of specific *Bifidobacterium* species, uniquely adapted to metabolize human HMOs [89]. Additionally, breast milk consumption has been variably associated with an increased abundance of *Lactobacillus* and reduced levels of Proteobacteria, *Veillonella*, Clostridium, and *Bacteroides*. However, these findings are not consistent across all studies [17,26,79]. Moreover, the exclusivity of breastfeeding (exclusive vs. partial breastfeeding) has a significant influence on infant gut microbiota composition, exhibiting clear dose-dependent relationships [28]. A meta-analysis conducted by Ho et al. [17] revealed higher relative abundances of genera such as *Eubacterium*, *Veillonella*, and *Bacteroides* in partially breastfed infants compared to those who were exclusively breastfed. Finally, cessation of breastfeeding has a pronounced influence on gut microbiota maturation, often exceeding the effect of introducing solid food, which rapidly shifts microbial communities toward a more diverse, adult-like state characterized by an increased abundance of Firmicutes [26,79,89,90].

Studies that extend the observational period beyond infancy (0–12 months) are limited, and it is rare to directly compare breastfed and weaned toddlers (1–3 years). Recently, Steward et al. conducted a longitudinal study (3–46 months) involving 903 children from six different geographical locations, including Finland, Germany, Sweden, and three U.S. states: Colorado, Georgia, and Washington [26]. They analyzed longitudinal fecal sample 16S rRNA sequence and metagenomic sequence and found that the receipt of breast milk was most significantly associated with *Bifidobacterium* throughout the 3–14 months, and breastfeeding had a comparable influence on microbiota development, regardless of whether it was exclusive or together with formula milk and/or solids [26]. In addition, their data showed that weaned infants and younger toddlers had significantly higher microbiota maturation than those who still received some breast milk, but this difference eventually converged by late toddlerhood [26]. Our findings largely align with theirs in terms of the general impact of breast milk on toddlers’ gut microbiota, emphasizing that continued breastfeeding into the toddler years prolongs the human-milk-associated *Bifidobacterium* and delays gut microbiota maturation.

Another large cohort longitudinal study regarding toddler gut microbiota was conducted by Bergström et al. in Denmark [79]. They collected samples at 9, 18, and 36 months and performed quantitative PCR to characterize 31 selected targeted bacteria 16S rRNA in the phylum Firmicutes, Bacteroidetes, Actinobacteria, Proteobacteria, Verrucomicrobia, and Euryarchaeota. Notably, the first four of them were identified as the most abundant phyla in our Peruvian cohort, and the first five were identified as the most abundant in the cohort studied by Steward et al. in the six Western developed countries mentioned earlier [26]. Both large-cohort longitudinal studies reported that the most dramatic microbiota transition occurs in late infancy and early toddlerhood, marked by a shift from a microbiota dominated by *Lactobacillus* (some genera now belong to the genus *Ligilactobacillus*), *Bifidobacterium*, and Enterobacteriaceae to one enriched with *Bacteroides* and some genera in Bacillota. We also observed similar genus-level shifts in the typical complementary feeding period.

In addition to aligning with the general patterns of gut microbiota maturation, our study provides new clarity to support the notion that microbial transition during toddlerhood is influenced by the timing of breastfeeding cessation, independently of the introduction of complementary foods. Prolonged breastfeeding is associated with a slower and steadier shift toward an adult-like microbiota in toddlers. However, the presence of human breast milk affects those key adult-associated bacterial genera in distinct ways. *Bacteroides*, a key beneficial genus involved in processing host- and plant-derived complex carbohydrates [91], maintained similar relative abundances in both BF and NBF toddlers throughout the second year of life, suggesting that its colonization is not significantly modulated by the presence of breast milk. *Faecalibacterium*, *Roseburia*, and *Prevotella*, which also utilize complex plant polysaccharides [92,93,94,95], were relatively less abundant in BF toddlers at 12 and 18 months of age, with levels becoming comparable to those of weaned peers by 24 months or later. These findings suggest that while the introduction of complementary foods promotes their growth, breastmilk components may delay their expansion. The HMOs selectively enhance the growth of *Bifidobacterium*, conferring competitive advantages that, in concert with breastmilk-derived antimicrobial proteins, create an environment less favorable for food-associated genera [96]. *Blautia*, another genus characteristic of adult-type microbiota [97], reached high and stable abundance rapidly in weaned toddlers and continued to increase among breastfed toddlers. It has been reported that *Blautia* is not suppressed by breast milk; instead, longer breastfeeding may be associated with a high abundance of *Blautia* [22,98].

It is also important to consider how geographical and dietary contexts might influence microbiota development. Our study focused on the children in a rural Peruvian region, where the complementary diets are rich in plant-based, high-fiber foods (e.g., yucca, bananas, local fruits), introduced as early as two months of age, alongside breastfeeding [31]. This may have contributed to certain observations, such as the notable increase in Prevotella in these children during late infancy and toddlerhood. *Prevotella* is a genus often associated with fiber-rich diets common in non-Western populations [80]. Its emergence in our cohort as the top abundant genus as early as 6 months old and its continued expansion in toddlerhood align with reports from other developing regions, where children consuming traditional diets harbor *Prevotella* as their microbiota matures [80]. In contrast, many infants in industrialized countries with lower dietary fiber intake at weaning have minimal *Prevotella* in the first years of life, instead showing greater abundances of *Bacteroides* when animal proteins and processed carbohydrates are introduced [99]. In our cohort, by two years old, children possessed more *Prevotella* than *Bacteroides* in both BF and NBF groups. Despite these differences, the overarching pattern holds; the cessation of breast milk selects for a broader array of microbes adapted to the new diet very fast, whereas continued breast milk maintains the earlier colonizers for longer and delays microbiota maturation.

### 4.3. Implications for Health and Development

The observed differences in gut microbiota between the BF and NBF toddlers may influence child health and development. The BF toddlers who continued to receive breast milk maintained higher levels of beneficial *Bifidobacterium* and other Actinobacteria through complementary feeding. *Bifidobacterium* species metabolize HMOs, resulting in the production of short-chain fatty acids (SCFAs) that lower gut pH, inhibit pathogens, and modulate immunity [18,96]. Extended breastfeeding may thus offer continued protection against infections and inflammatory diseases beyond infancy. Increased *Ligilactobacillus*, another beneficial lactic acid bacterium, in BF toddlers likely supports immune function and gut barrier integrity [100].

Conversely, the NBF toddlers showed earlier colonization by adult-associated taxa, including butyrate producers (*Faecalibacterium*, *Roseburia*) and fiber degraders (*Blautia*, *Prevotella*). *Faecalibacterium* and *Roseburia* potentially enhance gut mucosal health, immune regulation, and metabolic capacities earlier in life [101]. However, our study detected no overt health detriments associated with either microbiota pattern presented in the BF and NBF group by age two, and both trajectories appeared physiologically normal. It is worth noting that BF toddlers also eventually acquired adult-like taxa, indicating a gradual and potentially gentler microbial colonization process facilitated by prolonged breastfeeding.

While few studies address the microbiota implications of breastfeeding beyond 12 months, evidence from first-year breastfeeding indicates associated long-term physical and cognitive developmental benefits [102,103]. Our findings provide insight into this understudied aspect, demonstrating sustained bifidobacterial dominance and lower microbiota diversity in toddlers who continued to receive human breast milk.

### 4.4. Implications for Feeding Practices and Nutritional Guidance

From a public health and nutritional standpoint, our study provides a microbiological perspective for existing WHO feeding recommendations and suggests considerations for weaning strategies. Extended breastfeeding into the second year of life maintains beneficial microbiota (*Bifidobacterium*, *Ligilactobacillus*), enhancing immune protection and nutrient utilization during weaning. These findings provide caregivers and healthcare professionals with additional information when evaluating prolonged breastfeeding.

Regarding nutritional guidance, the timing of introducing complementary foods remains crucial. Early complementary feeding (<3 months) has been linked to increased infection risk and metabolic disorders, likely due to shortened breastfeeding duration [104]. Delayed complementary feeding may cause nutritional deficits and growth challenges [104,105]. In our cohort, most children had complementary solid food by 6 months, with the BF group showing similar, albeit delayed, microbiota responses to food. Our results imply the flexibility when implementing a weaning approach, in which prolonged breastfeeding allows gradual but sufficient microbiota adaptation to complementary foods, provided nutritional adequacy is maintained.

Lastly, our data may inform the development of breast milk substitutes containing prebiotics or probiotics, designed to replicate the microbiota profiles observed in extended breastfeeding in toddlerhood. However, further investigation into the health implications of sustaining beneficial microbial communities in toddlers remains essential.

### 4.5. Limitations

Our study has limitations. First, this was an observational analysis of an existing cohort (the Peruvian MAL-ED cohort). Although we rigorously controlled for available covariates, we cannot fully exclude the influence of unmeasured confounders, such as the type and amount of complementary food. Maternal decisions about how long to breastfeed may be linked to factors such as maternal health, nutrition, or socioeconomic status, which could independently affect the child’s microbiota. Secondly, our study design was partially longitudinal but not fully paired. Children contributed stool samples at set age windows, and not all had multiple samples over time. The dataset was unbalanced, e.g., relatively few BF children at 24 months, no NBF at 6 months, and relatively few NBF at 12 months due to natural weaning patterns. We treated each age group comparison essentially in a cross-sectional manner for BF vs. NBF, which could introduce some between-subject variability. As a result, some inter-individual differences may confound the effects of age or feeding, although our beta-diversity analyses and statistical models accounted for individual variation where possible. The cohort size and sampling frequency of MAL-ED are strengths. Still, future research with targeted longitudinal sampling around the weaning transition would be valuable to confirm our observations in a within-subject fashion. Third, the generalizability of some of our findings may be limited to similar contexts. The study children were from an Amazonian Peruvian community characterized by early introduction of foods, high rates of extended breastfeeding, and a diet relatively high in natural plant-based foods but low in processed foods. The microbiota succession we documented, including the significant presence of *Prevotella* and other anaerobes by the late secondary year, may differ in populations with different diets (e.g., higher animal protein or fat intake) or different environmental microbial exposures. Nonetheless, the fundamental biological phenomenon of breast milk altering the gut microbial trajectory is likely to hold broadly, even if the specific taxa involved vary. Fourth, the MAL-ED study collected samples up to the age of two years. Our study did not provide information on whether the observed impact of breastfeeding duration during the complementary feeding period, particularly in the second year of life, persisted long-term or lifelong. Additionally, our analysis focused on taxonomic composition based on 16S rRNA gene sequencing and did not directly assess microbiota function or strain-level differences. It is possible that BF and NBF toddlers harbored functionally distinct microbiomes even when taxonomic profiles began to converge.

## 5. Conclusions

In conclusion, this study provides compelling evidence that breastfeeding during the complementary feeding period (age of 6 to 24 months) has a significant impact on the trajectory of gut microbiota development in children under two years of age. While all toddlers eventually transition from a milk-oriented to a more adult-like microbiota, those who continue to receive breast milk alongside complementary foods do so on a delayed, more gradual path compared to those who are weaned. Prolonged breastfeeding maintained a bifidobacteria-rich, lower-diversity microbiota into the second year of life, whereas early weaning was associated with accelerated microbiota diversification and an earlier dominance of solid food-associated taxa. These findings highlight the significant impact of early childhood feeding practices on gut microbial ecology, with potential implications for nutrition, development, and overall health. They support current WHO recommendations that encourage sustained breastfeeding until two years or older and emphasize the importance of considering the microbiota as a factor in feeding decisions. Going forward, integrating microbiota-focused insights into child nutrition programs could help optimize health outcomes during this critical period of growth and development.

## Data Availability

The raw data presented in the study are openly available in European Nucleotide Archive (accession number PRJEB28159). Restrictions apply to the availability of the metadata. Metadata were obtained with permission from Clinical Epidemiology Resources (ClinEpiDB). The data generated and analyzed during the study will be made available by the corresponding author upon request.

## Supplementary Materials

The following supporting information can be downloaded at: https://www.mdpi.com/article/10.3390/ijerph22091369/s1, Table S1. Sample Distribution and Availability in the Study Population; Table S2. Sample Distribution by Age, Breastfeeding Status, and Gender; Microbial composition at the Phylum level between breastfeeding groups; Table S3. Statistics-Demographics related variables; Table S4. Statistics—Breastfeeding related variables; Table S5. Statistics—Complementary Food Introduction related variables; Table S6. Statistics -Vaccine related variables; Table S7. Statistics-Diarrheal related variables; Table S8. Statistics—Illnesses related variables; Figure S1. Sample Processing Diagram; Table S9A. Alpha Diversity Richness Statistics. Age affects the gut microbiome community; Table S9B. Alpha Diversity Richness Statistics. Breastfeeding status affects the gut microbiome community; Table S10A. Alpha Diversity Evenness Statistics. Age affects the gut microbiome community; Table S10B. Alpha Diversity Evenness Statistics. Breastfeeding status affects the gut microbiome community; Table S11A. Beta Diversity Statistics. Age affects the gut microbiome community; Table S11B. Beta Diversity Statistics. Breastfeeding status affects the gut microbiome community; Table S12A. Microbial composition at the Phylum level between age groups; Table S12B. Microbial composition at the Phylum level between breastfeeding groups; Table S13A. Microbial composition at the Genus level between age groups; Table S13B. Microbial composition at the Genus level between breastfeeding groups.

## Abbreviations

The following abbreviations are used in this manuscript:

BF	Breastfeeding group (Breastmilk + Complementary Foods)
NBF	Non-Breastfeeding group (Breastmilk Weaning Completed)
6M	6 months group
12M	12 months group
18M	18 months group
24M	24 months group
MAL-ED	Etiology, Risk Factors and Interactions of Enteric Infections and Malnutrition and the Consequences for Child Health and Development
ENA	European Nucleotide Archive
ClinEpiDB	Clinical Epidemiology Resources
rRNA	ribosomal RNA
ASVs	Amplicon Sequence Variants
OTUs	Operational Taxonomic Units
FDR	False Discovery Rate
PCoA	Principal Coordinates Analysis
PERMANOVA	Principal Coordinates Analysis
PERMDISP	Multivariate Dispersions
HMOs	Human Milk Oligosaccharides
SCFAs	Short-Chain Fatty Acids
WHO	World Health Organization
