# Supplementary material for: The Gut Microbiota of Peruvian Children Under the Age of Two During the Complementary Feeding Period
Source: Int J Environ Res Public Health. 2025 Aug 30;22(9):1369. doi: 10.3390/ijerph22091369 (PMC12469641; doi:10.3390/ijerph22091369)
Supplement: Supplementary file 1 [file ijerph-22-01369-s001.zip › ijerph-3507639-supplementary.pdf]

Supplementary Material

The Gut Microbiome of Peruvian Children under the Age of Two during the Complementary Feeding Period

Kutbi et al.

Table S1. Sample Distribution and Availability in the Study Population.

	**Availability of Samples per Participant**				**Gender**	
	**One sample**	**Two samples**	**Three samples**	**Four samples**	**Female**	**Male**
Number of Participant	44	54	103	60	119	142

Note: The data structure of the study is based on 261 participants. Samples were collected from these participants at intervals of 6, 12, 18 & 24 months of age. Samples availability per participant varies, ranging from one to four samples.

Table S2. Sample Distribution by Age, Breastfeeding Status, and Gender.

**Age**	**Breastfeeding (BF)**			**Non- Breastfeeding (NBF)**		
	**Samples**	**Female**	**Male**	**Samples**	**Female**	**Male**
6 months	213	93	120	-	-	-
12 months	180	86	94	7	2	5
18 months	103	42	61	77	36	41
24 months	23	12	11	98	49	49

Note: The data structure encompassing 701 samples, with 320 females and 381 males. The samples were collected at different time points (6, 12, 18, & 24 months), and the participants were either breastfeeding (BF) or not breastfeeding (NBF) at the time of sample collection.

Table S3. Statistics -Demographics related variables.

	12M			18M			24M		
	**BF** (n = 214)	**NBF** (n = 7)	**p**	**BF** (n = 113)	**NBF** (n = 83)	**p**	**BF** (n = 25)	**NBF** (n = 100)	**p**
Sex, N (%)<br>Female<br>Male	98 (45.8)<br>116(54.2)	2 (28.6)<br>5 (71.4)	0.4607	50 (44.2)<br>63 (55.8)	40 (48.2)<br>43 (51.8)	0.6637	13 (52)<br>12 (48)	51 (51)<br>49 (49)	1
Birth weight (kg), median (Q1, Q3)	3.11 (2.90, 3.36)	2.87 (2.84, 3.42)	0.6793	3.13 (2.90, 3.35)	3.08 (2.28, 3.39)	0.425	3.16 (3.05, 3.38)	3.09 (2.88, 3.30)	0.1116

Table S4. Statistics - Breastfeeding related variables.

	12M			18M			24M		
	**BF** (n = 214)	**NBF** (n = 7)	**p**	**BF** (n = 113)	**NBF** (n = 83)	**p**	**BF** (n = 25)	**NBF** (n = 100)	**p**
Age at complete weaning(days), median (Q1, Q3)	581 (497.0, 688.0)	279 (214.5, 318.5)	7.232 e-06	672 (595, 739.0)	474 (437, 511.5)	< 2.2 e-16	777.0 (757.0, 843.0)	551.5 (464.5, 647.5)	2.442 e-13
Fed colostrum, N (%)<br>No<br>Yes	7 (3.3)<br>207 (96.7)	0<br>7 (100)	1	3 (2.7)<br>110 (97.3)	3 (3.6)<br>80 (96.4)	0.6994	1 (4)<br>24 (96)	5 (5)<br>96 (95)	1
Prelacteal feeding, N (%)<br>No<br>Yes	195 (91.1)<br>19 (8.9)	7 (100)<br>0	1	103 (91.2)<br>10 (8.4)	76 (91.6)<br>7 (8.4)	1	24 (96)<br>1 (4)	91 (91)<br>9 (9)	0.6852
Time between childbirth and 1st breastfeeding, N (%)<br>Within 1 hour<br>1 hour to 24 hours<br>1 day to 3 days<br>4+ days	155(72.4)<br>52 (24.3)<br>4 (1.9)<br>3 (1.4)	7 (100)<br>0<br>0<br>0	0.3626	82 (72.6)<br>30 (26.5)<br>1 (0.9)<br>0	60 (72.3)<br>20 (24.1)<br>1 (1.2)<br>2 (2.4)	0.4699	17 (86)<br>8 (32)<br>0<br>0	74 (74)<br>23 (23)<br>1 (1)<br>2 (2)	0.7164

Breastfed within 1st 24hrs, N (%)<br>No<br>Yes	7 (3.3)<br>207 (96.7)	0<br>7 (100)	1	1 (0.9)<br>112 (00.1)	3 (3.6)<br>80 (96.4)	0.3129	0<br>25 (100)	3 (3)<br>97 (97)	1
Breastfeeding-Length, median (Q1, Q3)	366 (365.0, 367.0)	276 (185.5, 309.5)	5.867 e-06	549 (547.0, 550)	470 (436.5, 505)	< 2.2 e-16	730 (727, 731)	551 (463, 639)	1.899 e-12
Cumulative days exclusively breastfed, median (Q1, Q3)	97.5 (39.0, 150)	7.0 (4.5, 13)	0.0006809	113 (57, 157.0)	68 (27, 118.5)	0.0002801	59.0 (33.0, 113)	95.5 (31.5, 144)	0.5576
Cumulative days predominantly breastfed, median (Q1, Q3)	58 (21, 118)	55 (20, 114)	0.7823	60 (25.0, 106)	79 (32.5, 142)	0.07887	87 (47, 154)	65 (25, 125)	0.1855
Cumulative days partially breastfed, median (Q1, Q3)	184 (166, 212)	107 (64, 210)	0.09255	361 (339, 382.0)	300 (262, 342.5)	4.255 e-14	536.0 (514.0, 577.0)	368.5 (302.5, 451.5)	5.056 e-09
Cumulative days not breastfed, median (Q1, Q3)	0 (0, 0)	93 (57.5, 180)	< 2.2 e-16	0 (0, 0)	76 (43.5, 114)	< 2.2 e-16	0 (0, 3)	178.5 (92, 267)	1.85 e-12
Age last exclusively breastfed (days), median (Q1, Q3)	22.5 (7, 62.0)	5.0 (2, 7.5)	0.003813	25 (9.0, 85.0)	14 (4.5, 38.5)	0.006834	16 (9, 27.0)	18 (6, 65.5)	0.6389

Table S5. Statistics - Complementary Food Introduction related variables.

	12M			18M			24M		
	**BF** (n = 214)	**NBF** (n = 7)	**p**	**BF** (n = 113)	**NBF** (n = 83)	**p**	**BF** (n = 25)	**NBF** (n = 100)	**p**
Age 1st animal milk or formula given(days), median (Q1, Q3)	187 (82, 226)	45 (21, 129)	0.0936	195 (88, 228)	183 (57, 218)	0.3609	224 (85, 253)	186 (49, 226)	0.1536
Age 1st animal milk or solids given(days), median (Q1, Q3)	138 (45, 178)	45 (23, 128)	0.1887	151 (45, 128)	134 (45, 175)	0.1696	151 (45, 185)	136 (43, 178)	0.3886
Age 1st clear liquid given(days), median (Q1, Q3)	30 (10, 80)	7 (2, 10)	0.00489	37 (11, 111)	24 (7, 67)	0.03999	21 (10, 42)	26 (10, 82)	0.6938
Age 1st solid food given(days), median (Q1, Q3)	157 (117,182)	167 (154,175)	0.7033	165 (130,183)	152 (85, 178)	0.07208	165 (131,190)	157 (115,180)	0.3074

Table S6. Statistics -Vaccine related variables.

	12M			18M			24M		
	**BF** (n = 214)	**NBF** (n = 7)	**p**	**BF** (n = 113)	**NBF** (n = 83)	**p**	**BF** (n = 25)	**NBF** (n = 100)	**p**
Total yellow fever vaccine doses, N (%)<br>1	166 (100)	3 (100)	-	96 (100)	68 (100)	-	20 (100)	86 (100)	-
Total tetanus vaccine doses, N (%)<br>1<br>2<br>3<br>4<br>5	0<br>3 (1.4)<br>61 (28.5)<br>110 (51.4)<br>40 (18.7)	0<br>0<br>3 (42.9)<br>4 (57.1)<br>0	0.4997	0<br>1 (0.9)<br>27 (23.9)<br>63 (55.8)<br>22 (19.5)	0<br>1 (1.2)<br>18 (21.7)<br>48 (57.8)<br>16 (19.3)	0.9804	0<br>0<br>6 (24)<br>14 (56)<br>5 (20)	0<br>2 (2)<br>18 (18)<br>59 (59)<br>21 (21)	0.8595

Total rubella vaccine doses, N (%)<br>1<br>2<br>3	165 (85.1)<br>28 (14.4)<br>1 (0.5)	6 (100)<br>0<br>0	0.5014	86 (79.2)<br>21 (19.4)<br>1 (0.9)	68 (90.7)<br>7 (9.3)<br>0	0.06179	20 (83.3)<br>4 (16.7)<br>0	78 (83.9)<br>14 (15.1)<br>1 (1.1)	1
Total rotavirus vaccine doses, N (%)<br>1<br>2	2 (0.9)<br>211 (99.1)	0<br>7 (100)	1	1 (0.9)<br>112 (99.1)	1 (1.2)<br>80 (98.8)	0.3253	0<br>25 (100)	1 (1.0)<br>98 (99.0)	1
Total pneumococcal conjugate vaccine (PCV) doses, N (%)<br>1<br>2<br>3	3 (1.4)<br>22 (10.3)<br>189 (88.3)	0<br>1 (14.3)<br>6 (85.7)	0.589	1 (0.9)<br>5 (4.4)<br>107 (94.7)	1 (1.2)<br>10 (12.0)<br>72 (86.7)	0.09299	0<br>1 (4)<br>24 (96)	2 (2)<br>6 (6)<br>92 (92)	1
Total oral poliovirus vaccine (OPV) doses, N (%)<br>1<br>2<br>3<br>4<br>5	0<br>0<br>5 (2.3)<br>10 (4.7)<br>36 (16.8)	0<br>0<br>7 (100)<br>0<br>0	0.7622	0<br>2 (1.8)<br>78 (69.0)<br>6 (5.3)<br>27 (23.9)	0<br>2 (2.4)<br>68 (81.9)<br>2 (2.4)<br>11 (13.3)	0.1609	0<br>0<br>18 (72)<br>4 (16)<br>3 (12)	0<br>3 (3)<br>78 (78)<br>3 (3)<br>16 (16)	0.09867
Total mumps vaccine doses, N (%)<br>1<br>2	166 (86.0)<br>27 (14.0)	6 (100)<br>0	0.6402	86 (79.6)<br>22 (20.4)	68 (90.7)<br>7 (9.3)	0.04608	20 (83.3)<br>4 (16.7)	79 (84.9)<br>14 (15.1)	0.919
Total measles, mumps, and rubella (MMR) vaccine doses, N (%)<br>1<br>2<br>3	165 (85.1)<br>28 (14.4)<br>1 (0.5)	6 (100)<br>0<br>0	0.5014	86 (79.6)<br>21 (19.4)<br>1 (0.9)	68 (90.7)<br>7 (9.3)<br>0	0.06179	20 (83.3)<br>4 (16.7)<br>0	78 (83.9)<br>14 (15.1)<br>1 (1.1)	1
Total measles vaccine doses, N (%)<br>1<br>2<br>3	165 (85.1)<br>27 (13.9)<br>2 (1.0)	6 (100)<br>0<br>0	0.6537	86 (79.6)<br>21 (19.4)<br>1 (0.9)	68 (90.7)<br>6 (8.0)<br>1 (1.3)	0.05125	20 (83.3)<br>4 (16.7)<br>0	78 (83.9)<br>14 (15.1)<br>1 (1.1)	1

Total influenza vaccine doses, N (%)<br>1<br>2<br>3<br>4<br>5	23 (11.7)<br>87 (44.4)<br>53 (27.0)<br>28 (14.3)<br>5 (2.6)	1 (16.7)<br>4 (66.7)<br>1 (16.7)<br>0<br>0	0.7155	14 (13.1)<br>45 (42.1)<br>31 (29.0)<br>14 (13.1)<br>3 (2.8)	11 (14.5)<br>36 (47.4)<br>16 (21.1)<br>11 (14.5)<br>2 (2.6)	0.8046	4 (16.7)<br>7 (29.2)<br>9 (37.5)<br>2 (8.3)<br>2 (8.3)	11 (11.6)<br>37 (38.9)<br>27 (28.4)<br>18 (18.9)<br>2 (2.1)	0.3667
Total hepatitis B vaccine doses, N (%)<br>1<br>2<br>3<br>4	0<br>1 (0.5)<br>19 (8.9)<br>194 (90.7)	0<br>0<br>0<br>7 (100)	1	0<br>0<br>10 (8.8)<br>103 (91.2)	0<br>0<br>10 (12.0)<br>73 (88.0)	0.4831	0<br>0<br>1 (4)<br>24 (96)	0<br>0<br>11 (11)<br>89 (89)	0.4568
Total diphtheria, pertussis, and tetanus (DPT) vaccine doses, N (%)<br>1<br>2<br>3<br>4<br>5	0<br>3 (1.4)<br>61 (28.5)<br>110 (51.4)<br>40 (18.7)	0<br>0<br>3 (42.9)<br>4 (57.1)<br>0	0.4997	0<br>1 (0.9)<br>27 (23.9)<br>63 (55.8)<br>22 (19.5)	0<br>1 (1.2)<br>18 (21.7)<br>48 (57.8)<br>16 (19.3)	0.9804	0<br>0<br>6 (24)<br>14 (56)<br>5 (20)	0<br>2 (2)<br>18 (18)<br>59 (59)<br>21 (21)	0.8595
Total Haemophilus influenzae type B vaccine doses, N(%)<br>1<br>2<br>3<br>4<br>5	0<br>3 (1.4)<br>64 (29.9)<br>144 (67.3)<br>3 (1.4)	0<br>0<br>3 (42.9)<br>4 (57.1)<br>0	0.7367	0<br>1 (0.9)<br>29 (25.7)<br>80 (70.8)<br>3 (2.7)	0<br>1 (1.2)<br>20 (24.1)<br>62 (74.7)<br>0	0.5767	0<br>0<br>6 (24)<br>18 (72)<br>1 (4)	0<br>2 (2)<br>20 (20)<br>78 (78)<br>0	0.2704
Total Bacille Calmette-Guerin (BCG) vaccine doses, N (%) 1	212 (100)	7 (100)	-	111 (100)	83 (100)	-	25 (100)	99 (100)	-

Table S7. Statistics -Diarrheal related variables.

	12M			18M			24M		
	**BF** (n = 214)	**NBF** (n = 7)	**p**	**BF** (n = 113)	**NBF** (n = 83)	**p**	**BF** (n = 25)	**NBF** (n = 100)	**p**
Days since last diarrheal episode, median (Q1, Q3)	48 (21.0, 105)	54 (16.5, 123)	0.9948	51 (21, 89)	58 (19, 121)	0.2704	99 (52, 170.0)	99 (32, 200.5)	0.9083
Total diarrheal episodes, median (Q1, Q3)	9 (6.0, 14.0)	8 (6.5, 10.5)	0.6047	10 (6, 15)	9 (6, 12)	0.228	11 (8, 16)	9 (6, 14)	0.2148
Cumulative diarrheal episode count, median (Q1, Q3)	3 (2, 6)	3 (2, 4.5)	0.7852	6 (4.0, 9)	6 (3.5, 8)	0.3161	7 (6, 13)	7 (5, 10)	0.2756
Days between diarrheal episodes, median (Q1, Q3)	41.5 (18, 102)	31.5 (6, 56)	0.2702	53 (24, 119)	48 (22, 197)	0.6449	63.0 (23, 140)	63.5 (27, 129)	0.8339
Cumulative days within diarrheal episodes, median (Q1, Q3)	13 (6, 25)	15 (10.5, 22)	0.7984	23 (11, 35)	19 (13, 32)	0.4652	31 (16, 46)	25.5 (15, 38.5)	0.356
Total days in all diarrheal episodes, ex 1st days, median (Q1, Q3)	23 (13, 35)	26 (21, 35.5)	0.6436	24 (13, 39)	23 (14, 34)	0.375	25 (14, 36)	23.5 (14, 35.5)	0.3591

Total days in all diarrheal episodes, median (Q1, Q3)	32<br>(20, 47)	37<br>(27.5, 44.5)	0.8101	35<br>(20, 51)	32<br>(20.5, 45.5)	0.3271	39<br>(18.5, 46.5)	32<br>(18.5, 46.5)	0.2496
Stool mucus present<br>No<br>Yes	207 (96.7)<br>7 (3.3)	7 (100)<br>0	1	109 (96.5)<br>4 (3.5)	81 (97.6)<br>2 (2.4)	1	24 (100)<br>*missing =1	97 (97)<br>3 (3)	0.2825
Stool consistency<br>Formed<br>Soft<br>Watery	88 (41.1)<br>117 (54.7)<br>9 (4.2)	5 (71.4)<br>2 (28.6)<br>0	0.4374	59 (52.2)<br>53 (46.9)<br>1 (0.9)	43 (51.8)<br>40 (48.2)<br>0	1	15 (60)<br>10 (40)<br>0	56 (56)<br>41 (41)<br>3 (3)	1

Table S8. Statistics - Illnesses related variables.

	**12M**			**18M**			**24M**		
	**BF**<br>(n = 214)	**NBF**<br>(n = 7)	**p**	**BF**<br>(n = 113)	**NBF**<br>(n = 83)	**p**	**BF**<br>(n = 25)	**NBF**<br>(n = 100)	**p**
Total fever days, caregiver report, median (Q1, Q3)	33.5<br>(24, 48)	31<br>(18.5, 37.5)	0.32	38<br>(26, 53)	32<br>(24, 49)	0.2364	40<br>(29, 45)	35.5 (26.5, 52.5)	0.718
Total days with ALRI, median (Q1, Q3)	1<br>(0,2)	1<br>(1, 2.5)	0.2637	1<br>(0, 2)	1<br>(0, 2)	0.8714	1<br>(0, 2)	1<br>(0, 2)	0.3188
Total days in all ALRI episodes, median (Q1, Q3)	1<br>(0,2)	1<br>(1, 2.5)	0.3737	1<br>(0, 2)	1<br>(0, 2)	0.8323	1<br>(0, 2)	1<br>(0, 2)	0.3672
Total ALRI episodes, median (Q1, Q3)	1<br>(0,2)	1<br>(1, 2.5)	0.1471	1<br>(0, 2)	1<br>(0, 2)	0.9372	1<br>(0, 2)	1<br>(0, 2)	0.2194
Cumulative ALRI episode count, median (Q1, Q3)	0<br>(0, 1)	1<br>(0, 1.5)	0.09952	0<br>(0, 1)	0<br>(0, 1)	0.8153	1<br>(0, 2)	0.5<br>(0, 1)	0.1685

Cumulative days within ALRI episodes, median (Q1, Q3)	0 (0, 1)	1 (0, 1.5)	0.1643	0 (0, 1)	0 (0, 1)	0.6997	1 (0, 2)	0.5 (0, 2)	0.2407
Cumulative days with ALRI, median (Q1,Q3)	0 (0, 1)	1 (0, 1.5)	0.1444	0 (0, 1)	0 (0, 1)	0.7195	1 (0, 2)	0.5 (0, 2)	0.2224
Cumulative days of illness surveillance, median (Q1, Q3)	364 (357, 365)	362 (351, 364)	0.4553	545 (538, 547)	545 (534,547)	0.3069	726 (723, 729)	726 (706.5, 729)	0.3376
Age at last illness surveillance (days), median (Q1, Q3)	1102.5 (1008, 1122)	1108 (1087.5, 1118)	0.9258	1110 (1076, 1133)	1101 (1034, 1117)	0.04781	1109 (1079, 1123)	1109 (1083.5, 1123.5)	0.6302

Note: Note: Kruskal-Wallis tests were performed for continuous variables. Fisher's exact tests were performed for categorical variables.

Table S9A. Alpha Diversity Richness Statistics. Age affects the gut microbiome community.

Test Statistics	Breastfeeding (BF)						Non- Breastfeeding (NBF)		
	6M	12M		18M	24M		12m	18M	24M
Kruskal-Wallis p-value	2.2e-16						0.0001		
Kruskal-Wallis multiple comparisons/ Dunn's test	6M vs. 12M	6M vs. 18M	6M vs. 24M	12M vs. 18M	12M vs. 24M	18M vs. 24M	12M vs. 18M	12M vs. 24M	18M vs. 24M
q-value	3.75 e-29	5.47 e-44	3.66 e-24	2.89 e-05	1.76 e-06	1.33 e-02	0.0793	0.0037	0.0017

Table S9B. Alpha Diversity Richness Statistics. Breastfeeding status affects the gut microbiome community.

Test Statistics	12M		18M		24M	
	BF	NBF	BF	NBF	BF	NBF

Kruskal-Wallis p-value	0.0223	4.043e-07	0.0071

Table S10A. Alpha Diversity Evenness Statistics. Age affects the gut microbiome community.

Test Statistics	Breastfeeding (BF)						Non- Breastfeeding (NBF)		
	6M	12M	18M	24M			12m	18M	24M
Kruskal-Wallis p-value	2.2e-16						0.1551		
Kruskal-Wallis multiple comparisons/ Dunn's test	6M vs. 12M	6M vs. 18M	6M vs. 24M	12M vs. 18M	12M vs. 24M	18M vs. 24M	12M vs. 18M	12M vs. 24M	18M vs. 24M
q-value	2.16 e-16	2.81 e-30	1.06 e-19	2.23 e-05	2.72 e-07	5.46 e-03	-	-	-

Table S10B. Alpha Diversity Evenness Statistics. Breastfeeding status affects the gut microbiome community.

Test Statistics	12M		18M		24M	
	BF	NBF	BF	NBF	BF	NBF
Kruskal-Wallis p-value	0.0004		1.543e-08		0.8949	

Table S11A. Beta Diversity Statistics. Age affects the gut microbiome community.

Test Statistics	Breastfeeding (BF)						Non- Breastfeeding (NBF)		
	6M	12M		18M	24M		12m	18M	24M
PERMANOVA p-value	0.001						0.022		
PERMANOVA PAIRWISE	6M vs. 12M	6M vs. 18M	6M vs. 24M	12M vs. 18M	12M vs. 24M	18M vs. 24M	12M vs. 18M	12M vs. 24M	18M vs. 24M
q-value	0.0012	0.0012	0.0012	0.0012	0.0012	0.0110	0.136	0.039	0.136
PERMANOVA DISPERSION	6M vs. 12M	6M vs. 18M	6M vs. 24M	12M vs. 18M	12M vs. 24M	18M vs. 24M	12M vs. 18M	12M vs. 24M	18M vs. 24M
DISPERSION p-value	0.0001						0.3693		
DISPERSION PAIRWISE	6M vs. 12M	6M vs. 18M	6M vs. 24M	12M vs. 18M	12M vs. 24M	18M vs. 24M	12M vs. 18M	12M vs. 24M	18M vs. 24M
q-value	0.0002	0.0178	0.1222	0.9465	0.9917	0.9408	-	-	-

Table S11B. Beta Diversity Statistics. Breastfeeding status affects the gut microbiome community.

Test Statistics	12M		18M		24M	
	BF	NBF	BF	NBF	BF	NBF
PERMANOVA p-value	0.024		0.001		0.001	
DISPERSION p-value	0.2167		0.1045		0.4762	

Table S12A. Microbial composition at the Phylum level between age groups.

Phyla	Breastfeeding (BF)						Non- Breastfeeding (NBF)		
all age groups *	<2.2e-16						<2.2e-16		
between age groups **	6M vs. 12M	6M vs. 18M	6M vs. 24M	12M vs. 18M	12M vs. 24M	18M vs. 24M	12M vs. 18M	12M vs. 24M	18M vs. 24M
Actinomycetota	9.639 e-16	< 2.2 e-16	5.538 e-12	3.709 e-06	6.586 e-09	1.154 e-05	0.3438	0.0828	0.0145
Bacillota	2.2 e-16	< 2.2 e-16	4.879 e-13	2.102 e-06	6.736 e-09	0.0002	0.5332	0.2922	0.2661
Bacteroidota	5.657 e-16	< 2.2 e-16	1.488 e-09	0.0062	0.0010	0.1599	0.06865	0.0362	0.6717
Pseudomonadota	0.0416	0.0021	0.1263	0.1752	0.5616	0.8177	0.09079	0.1083	0.8829

Note: * Kruskal-Wallis test was employed to determine the significance (p-value) of all age groups based on breastfeeding status in terms of taxon composition. ** Kruskal-Wallis pairwise test was used to determine the significance (p-value) between age groups based on breastfeeding status in terms of taxon composition.

Table S12B. Microbial composition at the Phylum level between breastfeeding groups.

Phyla	12M		18M		24M	
	BF	NBF	BF	NBF	BF	NBF
Actinomycetota	0.0002		< 2.2e-16		4.047e-07	
Bacillota	0.0021		9.933e-12		0.0127	
Bacteroidota	0.8419		0.0380		0.6676	
Pseudomonadota	0.4171		0.3961		0.4756	

Note: Kruskal-Wallis pairwise test was used to determine the significance (p-value) between groups in terms of taxon composition.

Table S13A. Microbial composition at the Genus level between age groups.

Genera	Breastfeeding (BF)						Non- Breastfeeding (NBF)		
all age group *	< 2.2e-16						< 2.2e-16		
between age groups **	6M vs. 12M	6M vs. 18M	6M vs. 24M	12M vs. 18M	12M vs. 24M	18M vs. 24M	12M vs. 18M	12M vs. 24M	18M vs. 24M
Bacteroides	3.044 e-15	2.285 e-13	1.294 e-06	0.7312	0.5645	0.857	0.1763	0.1001	0.5935
Bifidobacterium	< 2.2 e-16	< 2.2 e-16	3.517 e-12	1.797 e-06	4.485 e-09	1.677 e-05	0.2537	0.0435	0.0099
Blautia	< 2.2 e-16	< 2.2 e-16	< 2.2 e-16	3.243 e-06	1.114 e-07	0.0018	0.8651	0.9693	0.6662
Escherichia	0.0487	2.013 e-05	0.0052	0.0091	0.0508	0.6789	0.1816	0.0973	0.3864
Faecalibacterium	< 2.2 e-16	< 2.2 e-16	< 2.2 e-16	1.838 e-09	1.252 e-08	0.0064	0.0346	0.0237	0.6136
Ligilactobacillus	< 2.2 e-16	< 2.2 e-16	1.595 e-14	0.0002	0.0358	0.9468	0.0432	0.0122	0.5105
Prevotella	1.257 e-12	< 2.2 e-16	< 2.2 e-16	1.539 e-07	2.356 e-06	0.0646	0.0340	0.0194	0.8183
Roseburia	7.512 e-06	< 2.2 e-16	< 2.2 e-16	4.7 e-12	1.224 e-13	0.0023	0.0565	0.0045	0.1735
Streptococcus	0.0024	1.792 e-09	0.0006	0.0009	0.0170	0.3107	0.0155	0.0023	0.0004

Note: * Kruskal-Wallis test was employed to determine the significance (p-value) of all age groups based on breastfeeding status in terms of taxon composition. ** Kruskal-Wallis pairwise test was used to determine the significance (p-value) between age groups based on breastfeeding status in terms of taxon composition.

Table S13B. Microbial composition at the Genus level between breastfeeding groups.

Genera	12M		18M		24M	
	BF	NBF	BF	NBF	BF	NBF
Bacteroides	0.1619		0.4286		0.3364	
Bifidobacterium	0.0002		< 2.2e-16		6.041e-07	
Blautia	0.0025		0.0004		0.687	
Escherichia	0.7704		0.2748		0.4392	
Faecalibacterium	0.0268		0.0003		0.6773	
Ligilactobacillus	0.0204		0.0107		0.0169	
Prevotella	0.8699		0.0149		0.9947	
Roseburia	1.709e-05		1.473e-10		0.0263	
Streptococcus	0.0079		0.0007		0.7864	

Note: Kruskal-Wallis pairwise test was used to determine the significance (p-value) between groups in terms of taxon composition.

Figure S1. Sample Processing Diagram.

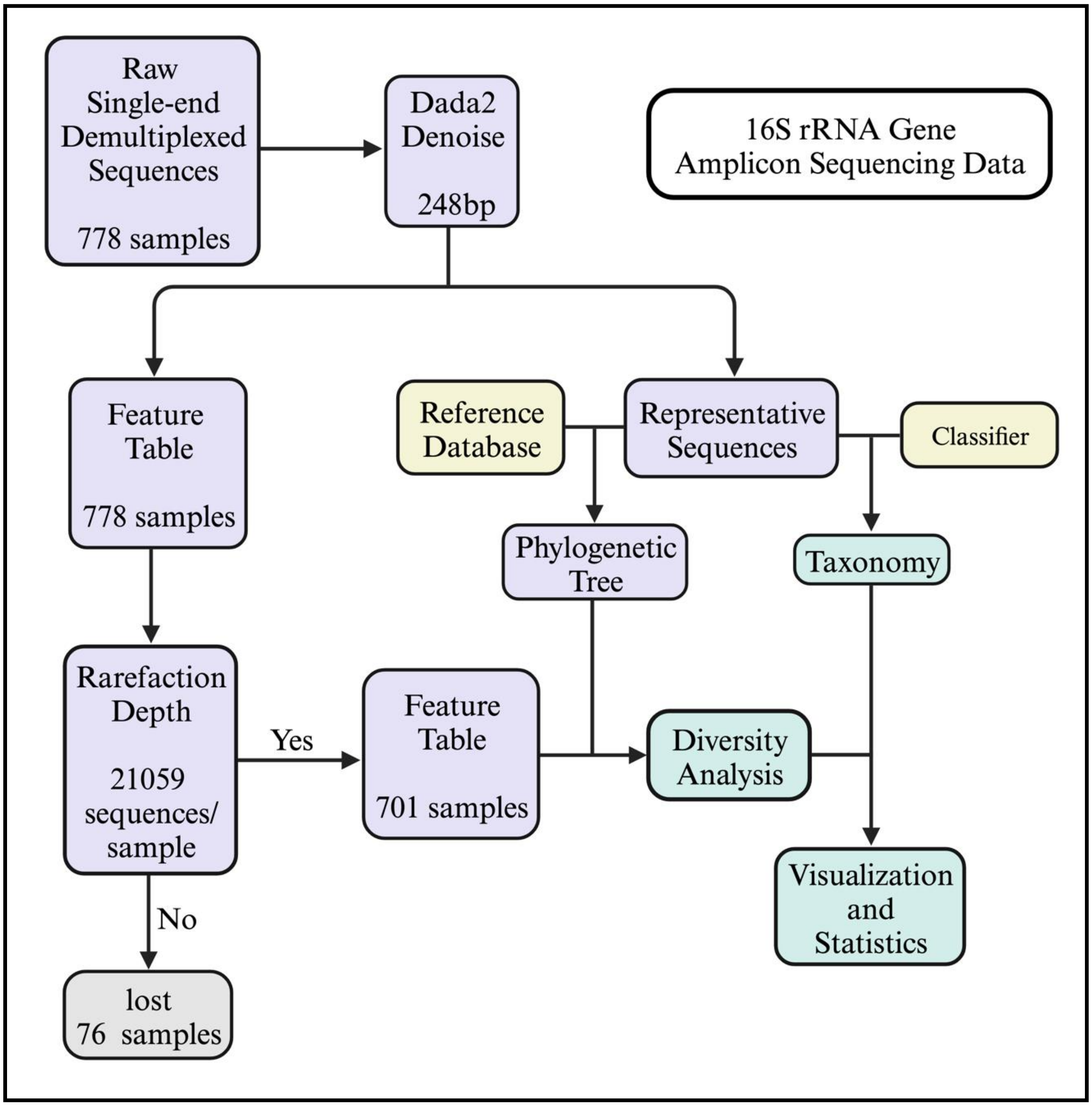

Note: The flowchart diagram illustrates the decrease in sample count from 778 to 701. At a sampling depth of 21059, 76 samples were lost. Additionally, one sample from the NBF group at 6 months was removed. As a result, there are 701 samples available for analysis.
